# The complete chloroplast genome sequence of *Cornus sunhangii* (Cornaceae)

**DOI:** 10.1080/23802359.2019.1669090

**Published:** 2019-09-25

**Authors:** Zhen-Yu Lv, Xian-Han Huang, Jian Luo, Xu Zhang, Tao Deng, Zhi-Min Li

**Affiliations:** a School of Life Sciences, Yunnan Normal University, Kunming, Yunnan, China;; b CAS Key Laboratory for Plant Diversity and Biogeography of East Asia, Kunming Institute of Botany, Chinese Academy of Sciences, Kunming, Yunnan, China;; c Tibet Agriculture & Animal Husbandry University, Nyingchi, Tibet, China;; d CAS Key Laboratory of Plant Germplasm Enhancement and Specialty Agriculture, Wuhan Botanical Garden, Chinese Academy of Sciences, Wuhan, Hubei, China

**Keywords:** *Cornus sunhangii*, chloroplast genome, phylogeny

## Abstract

*Cornus sunhangii* is a newly discovered species of Cornaceae and is endemic to Tibet, China. In this study, the complete chloroplast genome of *C. sunhangii* is described. The length of the chloroplast genome is 157,446 bp. The genome has quadripartite structure, which includes a large single-copy (LSC) region (86,861 bp), a small single-copy (SSC) region (18,331 bp), and a pair of inverted repeat (IR) regions (26,127 bp each). The complete chloroplast genome contains a total of 113 unique genes, of which 79 protein-coding genes, 30 tRNA genes, and 4 rRNA genes.

*Cornus sunhangii*, a newly described tree species by the authors recently, belongs to *Cornus* subg. *Syncarpea*, Cornaceae (Lv et al. 2019). It is endemic to the Mêdog, Tibet, China. *Cornus sunhangii* has potential economic benefits, because of similar species of *Cornus* having been used as food, oil, wood production, and ornament (Lv et al. 2019). However, its morphology is similar to *Cornus capitata* of *C.* subg. *Syncarpea* except for the fruit size. Therefore, we have assembled and reported the complete chloroplast genome of *C. sunhangii* to distinguish between *C. capitata* and *C. sunhangii*, and confirm phylogenetic status by maximum-likelihood (ML) method.

The plant samples were collected from north Mêdog County, Tibet, China (95.38000 E, 29.31806 N, 1988 m a.s.l) and deposited in the KUN (i.e. the herbarium of the Kunming Institute of Botany, CAS; holotype KUN1347138; isotypes KUN1347139, KUN1347140). In addition, the fresh leaves of *C. sunhangii* were collected for chloroplast DNA isolation. The genome was sequenced by Illumina HiSeq system at the Beijing Novogene Bioinformatics Technology Co., Ltd. (Nanjing, China). The reads were assembled using NOVOPlasty software (Dierckxsens et al. 2017). The chloroplast genome of *C. capitata* was selected as the reference genome. The annotation works of the genome were initially completed at GeSeq (Tillich et al. 2017), and then we referenced the result of DOGMA (Wyman et al. 2004) for manual correction with Geneious v.9.0.2 (Kearse et al. 2012). Finally, complete chloroplast genome of *C. sunhangii* was submitted to Genbank (Genbank Accession number is MN251844).

The length of the complete chloroplast genome of *C. sunhangii* is 157,446 bp and overall GC content is 38.1%. The genome is divided into four regions, which comprises a large single-copy (LSC) region (86,861 bp), a small single-copy (SSC) region (18,331 bp), and a pair of IR regions (26,127 bp each). The GC content of the IR regions (43.1%) is higher than of the SSC region (32.4%) and LSC region (36.3%). This genome contained a total of 113 unique genes, of which 79 were protein-coding genes, 30 tRNA genes, and 4 rRNA genes. Among these, 6 protein-coding genes (*rps12*, *rps7*, *ndhB*, *ycf2*, *rpl23*, and *rpl2*), 7 tRNA genes (*trnN-GUU*, *trnR-ACG*, *trnA-UGC*, *trnI-GAU*, *trnV-GAC*, *trnL-CAA*, and *trnH-CAU*), and 4 rRNA genes (*rrn5*, *rrn4.5*, *rrn23*, and *rrn16*) are duplicated in the IR regions.

Phylogenetic status of *C. sunhangii* was analyzed. The genomic data of all relevant species came from Genbank, and were aligned by MAFFT (Katoh et al. 2002). Conserved sequences were extracted using Gblock v.0.91b (Castresana 2000). Phylogenetic analysis was performed using ML method, which was executed in IQTREE v.1.6.10 (Trifinopoulos et al. 2016). The bootstrap support values were obtained from 1000 replicates. The ML tree consisted of 6 groups that were Curtisiaceae, Alangiaceae, Cornaceae, Hydrangeaceae, Mastixiaceae, and Nyssaceae, respectively, which were consistent with previous study (Fu et al. 2017). *Cornus sunhangii* was within clade of Cornaceae and closer to *Cornus capitata* with 100% BS value (Figure 1).

**Figure 1.**
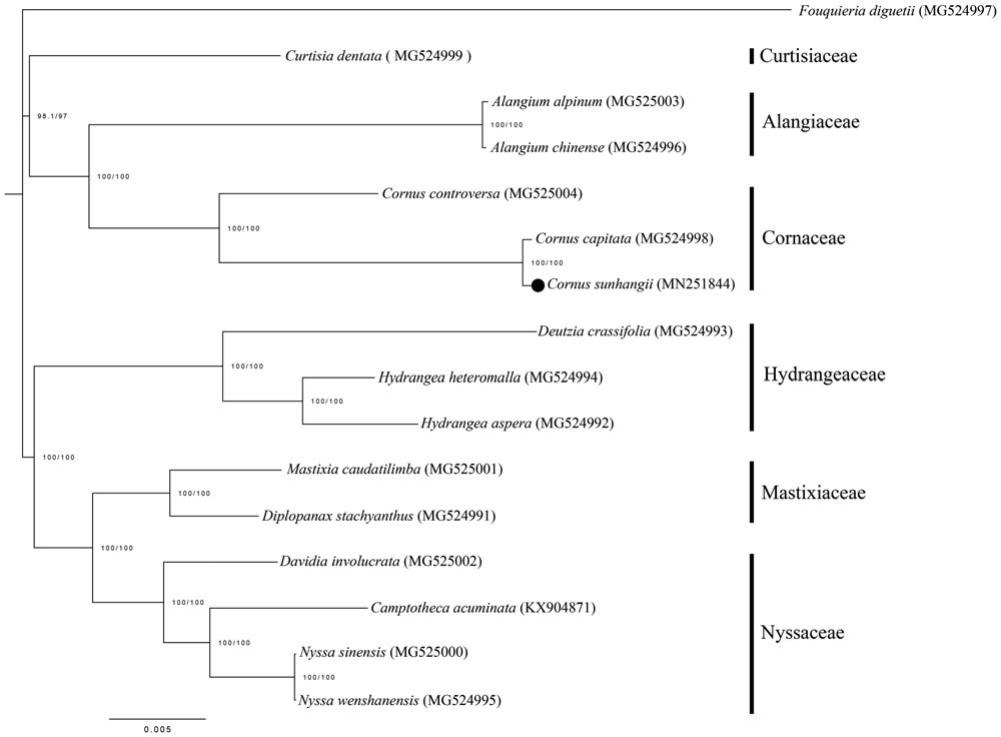
ML tree based on complete chloroplast genomes. The numbers are bootstrap values. The black dot indicates *Cornus sunhangii*.
